# Efficacy and safety of first- versus second-generation Bruton tyrosine kinase inhibitors in chronic lymphocytic leukemia: a systematic review and meta-analysis

**DOI:** 10.3389/fphar.2024.1413985

**Published:** 2024-07-10

**Authors:** Liyuan Ke, Su Li, Danxue Huang, Yan Wang

**Affiliations:** Department of Pharmacy, Cancer Hospital of China Medical University, Liaoning Cancer Hospital & Institute, Shenyang, China

**Keywords:** Bruton tyrosine kinase inhibitor, chronic lymphocytic leukemia, efficacy, safety, meta-analysis

## Abstract

We conducted this first systematic review and meta-analysis to assess the competitive advantage of 2nd-generation Bruton tyrosine kinase inhibitors (BTKi) compared to 1st-generation BTKi in chronic lymphocytic leukemia (CLL). The literature search was conducted from PubMed, Web of Science, Embase databases, and hematology annual conferences. Data of over response rate (ORR), progression-free survival (PFS), and overall survival (OS) were extracted to a pool meta-analysis of efficacy; adverse events (AEs) were also extracted to a pool meta-analysis of safety. Bias risk assessment and meta-analysis were performed by Review Manager 5.3 and STATA 14 software. A total of 3649 patients from 29 cohorts were included. The results showed that the benefits of ORR and 24-month PFS in 2nd-generation BTKi compared to 1st-generation BTKi were not significant in the whole population but only in the relapsed or refractory (R/R) CLL patient subgroup (ORR: 86.4% vs. 76.2%, *p* = 0.013; 24-month PFS: 76.9% vs. 67.9%, *p* = 0.004). Any-grade AEs were comparable between 1st- and 2nd-generation BTKi, but grade 3 or higher AEs were significantly less frequent with 2nd-generation BTKi *versus* 1st-generation BTKi (grade 3 or higher: 53.1% vs. 72.5%; *p* = 0.002). Headache was more frequent with 2nd-generation BTKi, while diarrhea and atrial fibrillation were more frequent with 1st-generation BTKi. Only for patients with relapsed or refractory CLL did 2nd-generation BTKi have a competitive advantage, while adverse effects still need to be considered.

**Systematic Review Registration:** https://www.crd.york.ac.uk/PROSPERO, Identifier 42022342488.

## 1 Introduction

Chronic lymphocytic leukemia (CLL) is the most common type of adult leukemia. According to cancer statistics published by the American Cancer Society, 20,160 new cases and 4,410 deaths from CLL were estimated in 2022 (Siegel et al., 2022). Although CLL is difficult to cure and often requires repeated treatment, durable remission can be achieved (Shadman, 2023). With the development of pathological signaling pathways in CLL, the standard of care for treating CLL is no longer limited to chemoimmunotherapy (Hallek et al., 2018). The B-cell receptor pathway is actively involved in the proliferation and survival of cancer cells, and Bruton tyrosine kinase (BTK) is an important component of this signaling pathway (Burger and Wiestner, 2018). Over the last few years, covalent and non-covalent (pirtobrutinib) BTK inhibitors (BTKi) have been approved for the treatment of CLL. Considering that pirtobrutinib was designed for R/R patients who had previously received a BTKi, this review only focuses on covalent BTKi. First-generation covalent BTKi include ibrutinib, while second-generation covalent BTKi include acalabrutinib, zanubrutinib, tirabrutinib, and orelabrutinib. First-generation BTKi showed advantages over standard chemoimmunotherapy but was limited by cardiovascular side effects. Second-generation BTKis were more selective and showed reduced rates of cardiovascular complications.

Ibrutinib, a 1st-generation irreversible BTKi, changed the treatment landscape of CLL. Based on the benefit of ibrutinib in clinical studies, clinical practice guidelines for CLL recommended ibrutinib as standard therapy for both treatment-naive (TN) and R/R patients (Eichhorst et al., 2021; Wierda et al., 2022). However, treatment discontinuation resulting from off-target binding has limited the use of ibrutinib because of atrial fibrillation, infections, and hemorrhage (Estupiñán et al., 2021). In order to avoid off-target binding, 2nd-generation BTKi with greater selectivity and fewer side effects was developed (Danilov et al., 2020; Byrd et al., 2021a; Tam et al., 2022; Xu et al., 2023). The ELEVATE-TN and ASCEND studies demonstrated that acalabrutinib provided superior PFS to chemoimmunotherapy in TN and R/R CLL, respectively (Ghia et al., 2020; Sharman et al., 2022). Zanubrutinib improved PFS for untreated CLL patients *versus* bendamustine–rituximab in the SEQUOIA trial (Tam et al., 2022).

In phase Ⅲ randomized controlled studies with ibrutinib as a control group, zanubrutinib demonstrated significantly longer PFS, while acalabrutinib only showed non-inferior PFS (Byrd et al., 2021b; Brown et al., 2023). The participants enrolled in these head-to-head trials were those with relapsed or refractory CLL. It is unclear whether there is a significant advantage of 2nd-generation over 1st-generation BTKi for patients with R/R CLL and whether this advantage yet persists in patients with TN CLL. There are also limited data that compare the safety of 1st- and 2nd-generation BTKi.

We conducted this systemic review and meta-analysis to assess the efficacy and safety differences between 1st- and 2nd-generation BTKi for untreated and relapsed or refractory CLL.

## 2 Methods

This study followed the Preferred Reporting Items for Systematic Reviews and Meta-Analyses (PRISMA) reporting guidelines (Liberati et al., 2009).

### 2.1 Search strategy

An initial systematic search was conducted on the PubMed, Web of Science, and Embase databases on 30 October 2023. A further manual search was executed by consulting references in relevant articles and abstracts of annual meetings published by hematologic oncology academic societies from 2015 to 2022, including the American Society of Hematology (ASH), the European School of Hematology (ESH), the American Society of Clinical Oncology (ASCO), and the European Society for Medical Oncology (ESMO). The full search algorithm is presented in Supplementary Table S1.

### 2.2 Selection criteria

The inclusion criteria include the following. 1) The clinical studies should be prospective trials to evaluate the efficacy and safety of BTKi used as monotherapy. 2) BTKi included 1st-generation inhibitor ibrutinib and 2nd-generation inhibitors acalabrutinib, zanubrutinib, orelabrutinib, and tirabrutinib. 3) Patients were diagnosed with CLL as defined by International Workshop on Chronic Lymphocytic Leukemia or World Health Organization criteria. 4) At least one of the following efficacy and safety data were reported: survival data measured by OS and PFS; overall response specified as complete response, partial response, and partial response with lymphocytosis; safety data containing any-grade AEs; grade 3 or high AEs; common AEs; AEs of special interest; AEs leading to treatment discontinuation. 5) There was no restriction on trial phase, dosing schedule, geographic region, refractory status, or mutant status. 6) The articles were written in English.

The exclusion criteria included retrospective studies, animal studies, review articles, case reports, repeated reports of the same trial, and trials with fewer than 20 participants.

### 2.3 Screening process

Two researchers consolidated the articles, searched from all sources, and removed duplicates. They scanned the titles and abstracts and then initially screened the articles according to the inclusion and exclusion criteria. After initial screening, suitable articles were subject to the re-screening process of the full text review. All the screening processes were completed independently by two researchers. Two researchers ultimately collated the screening results, and their disputes were resolved by the third researcher.

### 2.4 Data extraction

The articles included were identified by the first author’s last name plus the year of publication. If two articles had the same identifier, the first author’s full name was recorded to distinguish them. If there were two eligible trial cohorts in the same article, we marked them with A and B. The demographic and clinical characteristics of patients and the basic information of the clinical trial were extracted and detailed in the standard table. The extracted data included trial phase, ClinicalTrials.gov number, sample size, region, age, sex, intervention, dosage, previous treatment, Eastern Cooperative Oncology Group (ECOG) scores, outcomes, and median follow-up time.

### 2.5 Bias risk assessment

According to the study design, the included studies were divided into randomized and non-randomized trials for bias risk assessment. The Cochrane collaboration tool was used to assess risk of bias in randomized trials, and the MINORS instrument was used to assess risk of bias in non-randomized studies (Slim et al., 2003; Higgins et al., 2011). The Cochrane collaboration tool covered six domains of bias: selection, performance, detection, attrition, reporting, and other biases. We assessed the risk of bias from six domains in each randomized trial and plotted a graph using Review Manager 5.3. The revised version of MINORS included eight items; each was scored from 0 to 2. We recorded the score of each item and the total score in a table.

### 2.6 Data analysis

Meta-analysis was performed using STATA 14 software. Meta-analysis of efficacy was conducted to pool ORR, PFS, and OS across 1st-generation BTKi and 2nd-generation BTKi. Meta-analysis of safety was conducted to pool any-grade AEs, grade 3 or higher AEs, common AEs, AEs of special interest, and AEs leading to treatment discontinuation. Heterogeneity was measured by I^2^ and *p*-value. A fixed-effects model was used when heterogeneity was low (I^2^ <50% or *p*-value > 0.1). Otherwise, the random-effects model was used.

## 3 Results

A total of 820 articles were searched, including 817 from databases and three from manual searching. After removing duplicate articles, animal studies, review articles, and case reports, we collected 394 articles to enter the screening stage. According to inclusion and exclusion criteria, 24 articles were finally included in the meta-analysis through two-step screening. The screening flow diagram is shown in Supplementary Figure S1.

Finally, a total of 29 trial cohorts from 24 studies were included in our meta-analysis. Among them, 1643 patients from 12 cohorts were treated with 1st-generation BTKi, and 2006 patients from 17 cohorts were treated with 2nd-generation BTKi. The trial cohorts treated with 2nd-generation BTKi included ten cohorts receiving acalabrutinib, four receiving zanubrutinib, two receiving tirabrutinib, and only one cohort receiving orelabrutinib. In the 2nd-generation BTKi studies, there were six trial cohorts from phase Ⅲ clinical studies and 11 trial cohorts from phase Ⅱ or Ⅰ clinical studies. Among the 12 trial cohorts receiving 1st-generation BTKi treatment, seven were from phase Ⅲ clinical studies and five were from phase Ⅱ or Ⅰ clinical studies.

In the studies of 1st-generation BTKi, most patients had recurrent or refractory CLL, accounting for 75.7% (*n* = 1244), while 24.3% (*n* = 399) of patients received initial treatment. Like the distribution of patients receiving 1st-generation BTKi, 66.7% (*n* = 1338) R/R patients and 33.3% (*n* = 668) TN patients constituted the population receiving 2nd-generation BTKi. All studies reported AE, and all but one reported ORR. Survival data were reported in 25 trial cohorts. The median follow-up time of the studies included varied from 9.4 to 87 months, with 14 studies having a median follow-up time of more than 24 months. Details of the included studies are listed in Table 1. Other demographic and clinical characteristics are summarized in Supplementary Table S2.

**TABLE 1:** Characteristics of included studies.

Study ID	Trial phase	ClinicalTrials. gov identifier	Sample size	Intervention	Previous treatment	Reported outcomes	Median follow-up time, months
Second-generation BTK inhibitor							
(Byrd et al., 2021b)	Ⅲ	NCT02477696	268	Acalabrutinib	R/R	ORR, PFS, OS, and AE	40.9 (0.0–59.1)
(Sharman et al., 2020)	Ⅲ	NCT02475681	179	Acalabrutinib	TN	ORR, PFS, OS, and AE	28.3
(Ghia et al., 2020)	Ⅲ	NCT02970318	155	Acalabrutinib	R/R	ORR, PFS, OS, and AE	16.1
(Rogers et al., 2021)	Ⅱ	NCT02717611	60	Acalabrutinib	R/R	ORR, PFS, OS, and AE	35
(Sun et al., 2020)	Ⅱ	NCT02337829	48 (R/R:32, TN:16)	Acalabrutinib	TN, R/R	ORR, PFS, and AE	-
(Byrd et al., 2020a)	Ⅰb/Ⅱ	NCT02029443	134	Acalabrutinib	R/R	ORR, PFS, and AE	41 (0.2–58)
(Byrd et al., 2021a)	Ⅰ/Ⅱ	NCT02029443	99	Acalabrutinib	TN	ORR, PFS, and AE	53 (1–59)
(Cull et al., 2022)	Ⅰ/Ⅱ	NCT02343120	101	Acalabrutinib	R/R	ORR, PFS, OS, and AE	54.1
(Cull et al., 2022)	Ⅰ/Ⅱ	NCT02343120	22	Acalabrutinib	TN	ORR, PFS, OS, and AE	54.1
(Awan et al., 2019)	Ⅰ/Ⅱ	NCT02029443	33	Acalabrutinib	R/R	ORR, PFS, and AE	19.0 (0.7–30.6)
(Brown et al., 2023)	Ⅲ	NCT03734016	327	Zanubrutinib	R/R	ORR, PFS, OS, and AE	29.6
(Tam et al., 2022)	Ⅲ	NCT03336333	241 (without del17)	Zanubrutinib	TN	ORR, PFS, OS, AE	26.2
(Tam et al., 2022)	Ⅲ	NCT03336333	111 (with del17)	Zanubrutinib	TN	ORR, PFS, OS, and AE	26.2
(Xu et al., 2020)	Ⅱ	NCT03206918	91	Zanubrutinib	R/R	ORR, PFS, and AE	15.1 (0.8–21.2)
(Danilov et al., 2020)	Ⅰb	NCT02457598	29	Tirabrutinib	R/R	ORR and AE	15.5
(Walter et al., 2016)	Ⅰ	NCT01659255	28	Tirabrutinib	R/R	ORR and AE	-
(Xu et al., 2023)	Ⅱ	NCT03493217	80	Orelabrutinib	R/R	ORR, PFS, OS, and AE	32.3
First-generation BTK inhibitor							
(Brown et al., 2023)	Ⅲ	NCT03734016	325	Ibrutinib	R/R	ORR, PFS, OS, and AE	29.6
(Langerbeins et al., 2022)	Ⅲ	NCT02863718	182	Ibrutinib	TN	AE	31
(Byrd et al., 2021b)	Ⅲ	NCT02477696	265	Ibrutinib	R/R	ORR, PFS, OS, and AE	40.9 (0.0–59.1)
(Sharman et al., 2021)	Ⅲ	NCT02301156	62	Ibrutinib	R/R	ORR and AE	41.6
(Burger et al., 2020)	Ⅲ	NCT01722487	136	Ibrutinib	TN	ORR, PFS, OS, and AE	60
(Huang et al., 2018)	Ⅲ	NCT01973387	106	Ibrutinib	R/R	ORR, PFS, OS, and AE	17.8
(Byrd et al., 2014)	Ⅲ	NCT01578707	195	Ibrutinib	R/R	ORR, PFS, OS, and AE	9.4
(Burger et al., 2019)	Ⅱ	NCT02007044	104 (R/R:89, TN:15)	Ibrutinib	TN, R/R	ORR, PFS, OS, and AE	36
(Farooqui et al., 2015)	Ⅱ	NCT01500733	51 (R/R:16, TN:35)	Ibrutinib	TN, R/R	ORR, PFS, and AE	24
(Byrd et al., 2020b)	Ⅰb/Ⅱ	NCT01105247,NCT01109069	101	Ibrutinib	R/R	ORR, PFS, OS, and AE	87
(Byrd et al., 2020b)	Ⅰb/Ⅱ	NCT01105247,NCT01109069	31	Ibrutinib	TN	ORR, PFS, OS, and AE	87
(Byrd et al., 2013)	Ⅰb/Ⅱ	NCT01105247	85	Ibrutinib	R/R	ORR, PFS, OS, and AE	20.9

R/R, relapsed or refractory; TN, treated naive; ORR, over response rate; PFS, progression-free survival; OS, overall survival; AE, adverse event.

The 11 randomized trials were evaluated by the Cochrane collaboration tool and demonstrated low risk of bias, except for performance bias. Most of the included randomized studies were open-label trials, so the participants and personnel were not blinded; only the independent review committee assessed the response in a blinded manner. This resulted in a high risk of performance bias. The risk of bias is presented by traffic light and bar plot (Supplementary Figure S2). Utilizing the MINORS instrument, we evaluated the overall score of the non-controlled trials to be more than 12 points, suggesting a low risk of bias. The scores for each non-randomized trial on MINORS item are listed in Supplementary Table S3.

**FIGURE 1:**
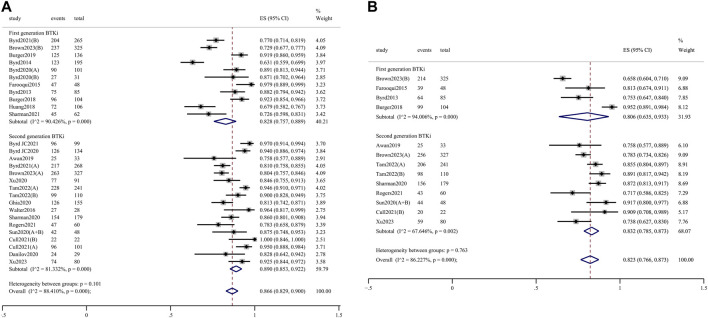
Forest plots for the pooled overall response rate **(A)** and 24-month progression-free survival **(B)** in all populations.

**FIGURE 2:**
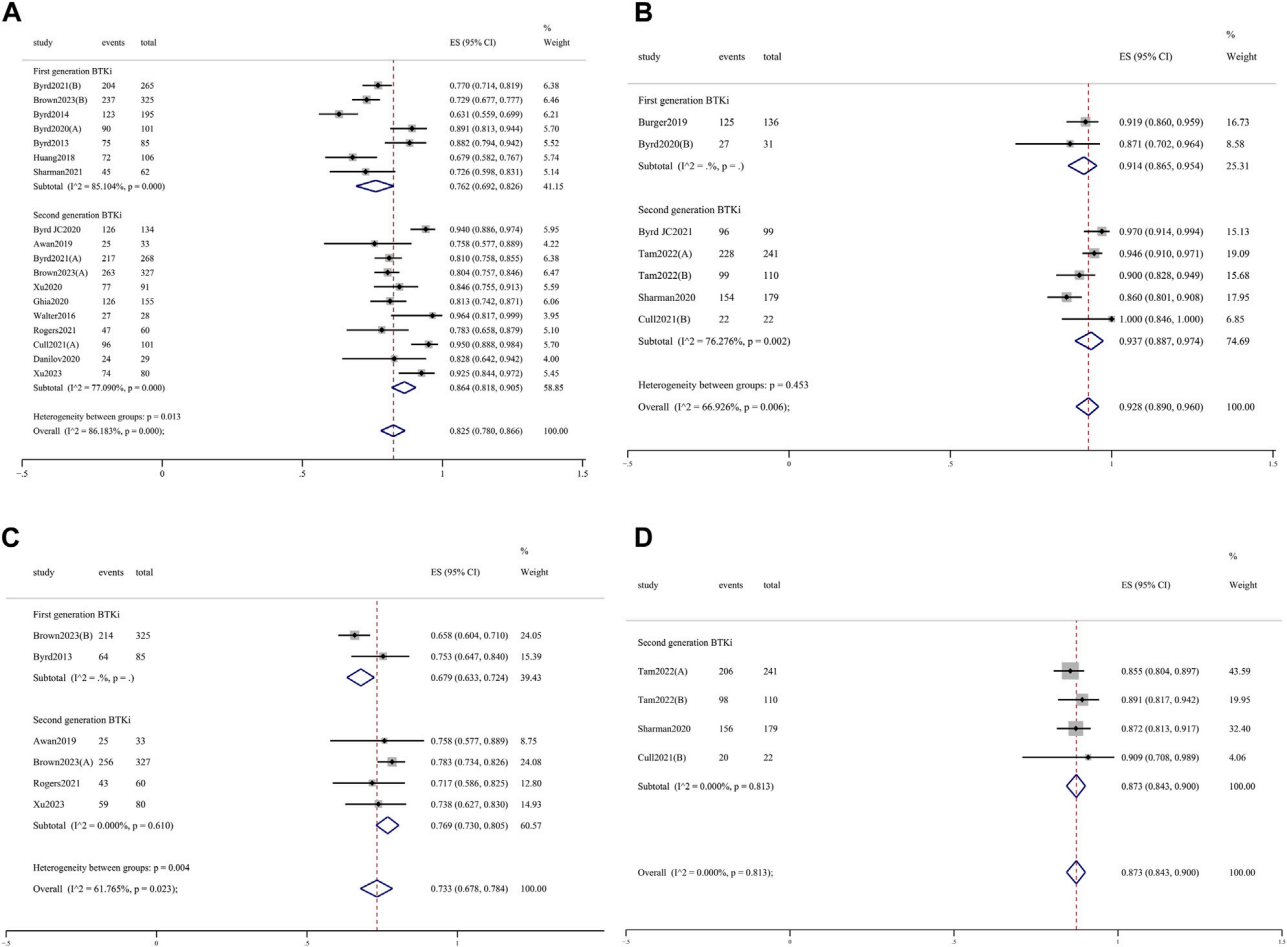
Forest plots for the pooled overall response rate in R/R **(A)** and TN **(B)** patients and 24-month progression-free survival in R/R **(C)** and TN **(D)** patients.

The pooled ORR for all trial cohorts was 86.6% (95% CI 82.9%–90.0%) (Figure 1A). The pooled ORR for cohorts treated with 2nd-generation BTKi was higher than that of 1st-generation BTKi, although the difference was not significant (89.0%, 95% CI 85.3%–92.2% vs. 82.8%, 95% CI 75.7%–88.9%; *p* = 0.101). In the R/R patient subgroup, the pooled ORR for 2nd-generation BTKi was significantly higher than for 1st-generation BTKi (86.4%, 95% CI 81.8%–90.5% vs. 76.2%, 95% CI 69.2%–82.6%; *p* = 0.013) (Figure 2A). In the subgroup analysis of TN patients, the pooled ORRs for 1st- and 2nd-generation BTKis were 91.4% (95% CI 86.5%–95.4%) and 93.7% (95% CI 88.7%–97.4%), respectively (Figure 2B). The pooled rate of 24-month PFS was 82.3% (95% CI 76.6%–87.3%), and that for 1st- and 2nd-generation were 80.6% (95% CI 63.5%–93.3%) and 83.2% (95% CI 78.5%–87.3%), respectively (Figure 1B). In the R/R patient subgroup, the pooled rate of 24-month PFS for patients treated with 2nd-generation BTKi was higher than that for 1st-generation BTKi with a significant difference (76.9%, 95% CI 73.0%–80.5% vs. 67.9%, 95% CI 63.3%–72.4%; *p* = 0.004) (Figure 2C). Notably, the pooled rate of 24-month PFS for TN patients treated with 2nd-generation BTKi was as high as 87.3% (95% CI 84.3%–90.0%) (Figure 2D). A total of 1171 patients reported 24-month OS data. The pooled rate of 24-month OS was 90.5% (95% CI 85.6%–94.6%), which was similar between the two generations of BTKis. Compared to the general population, the pooled rate of 24-month OS was slightly higher for TN patients while lower for R/R patients. No matter the status of previous treatment, the difference of 24-month OS between patients treated with 1st- and 2nd-generation BTKi was not significant. Details are listed in Supplementary Figure S3.

Nine of the included articles reported long-term survival data, including the rates of OS and PFS at 36 months, 48 months, 60 months, and up to 84 months. Long-term survival reports after 24 months were inconsistent across these studies. For 1st-generation BTKi, the rates of 36-month OS and PFS were 74% and 59%, respectively, in Byrd’s study, while they were up to 92% and 86% in Burger’s study. For 2nd-generation BTKi, two studies reported the rates of 36-month OS as 78.3% and 91%, and the rates of 36-month PFS were 58.3% and 83%. For 1st-generation BTKi, the rates of 60-month OS ranged 60%–92%, and the rates of 60-month PFS ranged 40%–92%. The longest follow-up time in the aggregated data was 84 months, at which the rate of OS was still up to 84% and the rate of PFS was 83%. The long-term survival data beyond 48 months for the 2nd-generation BTKi are currently lacking. Supplementary Table S4 gives details.

**FIGURE 3:**
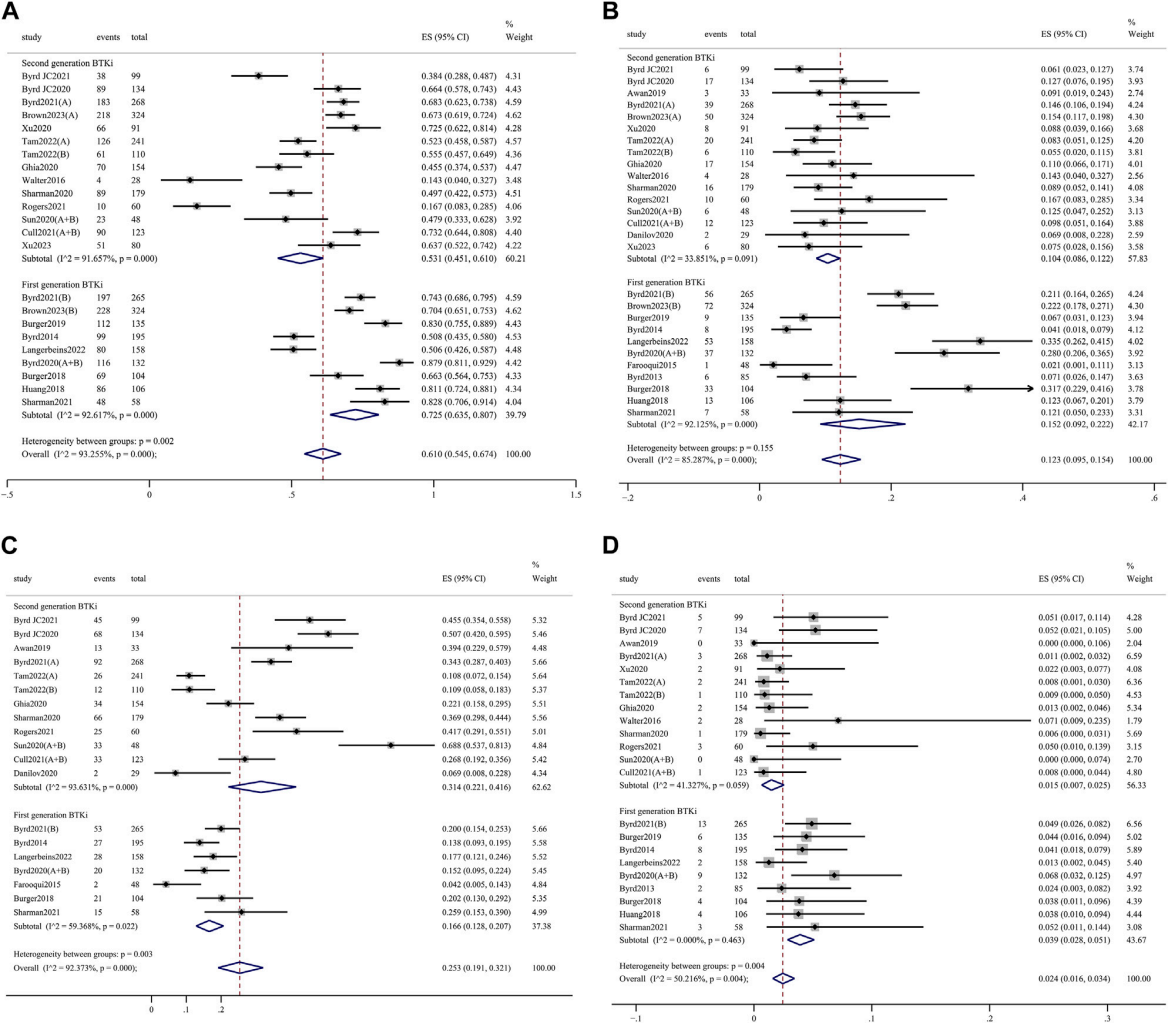
Forest plots for pooled incidences of grade 3 or higher adverse events **(A)**, treatment discontinuation **(B)**, any-grade headache **(C)**, and grade 3 or high diarrhea **(D)**.

Any-grade AEs were comparable between 1st- and 2nd-generation BTKi (98.8%, 95% CI 97.4%–99.8% vs. 98.6%, 95% CI 97.0%–99.6%; *p* = 0.629) (Supplementary Figure S4). Grade 3 or higher AEs were significantly less frequent for 2nd-generation BTKi *versus* 1st-generation BTKi (53.1%, 95% CI 45.1%–61.0% vs. 72.5%, 95% CI 63.5%–80.7%; *p* = 0.002) (Figure 3A). In the meta-analysis of discontinuation, AEs led to treatment discontinuation in 10.4% (95% CI 8.6%–12.2%) of patients treated with 2nd-generation BTKi and 15.2% (95% CI 9.2%–22.2%) treated with 1st-generation BTKi (Figure 3B). The most common AEs in the collected data included headache, diarrhea, cough, arthralgia/myalgia, neutropenia, and upper respiratory tract infections. Any-grade headache was less frequent with 1st-generation BTKi *versus* 2nd-generation BTKi (16.6%, 95% CI 12.8%–20.7% vs. 31.4%, 95% CI 22.1%–41.6%; *p* = 0.003) (Figure 3C). The incidence of grade 3 or higher diarrhea was higher in the 1st-generation BTKi group than in the 2nd-generation BTKi group (3.9%, 95% CI 2.8%–5.1% vs. 1.5%, 95% CI 0.7%–2.5%; *p* = 0.004) (Figure 3D). The incidence of any-grade diarrhea was higher for 1st-generation BTKi than 2nd-generation BTKi, with no statistically significant difference (39.5%, 95% CI 33.0%–46.2% vs. 32.3%, 95% CI 24.7%–40.4%; *p* = 0.178). The incidence of any-grade cough was similar between the two generations of BTKi. The incidences of any-grade neutropenia, arthralgia/myalgia, and upper respiratory tract infections were lower in the 1st-generation BTKi group than in the 2nd-generation group. For grade 3 or higher AEs, a lower incidence of neutropenia was reported in 1st-generation BTKi group than in the 2nd-generation group, with no statistically significant difference (14.8%, 95% CI 12.2%–17.7% vs. 16.3%, 95% CI 12.7%–20.1%; *p* = 0.515). In addition, the other incidences of grade 3 or higher AEs were extremely low. Details are listed in Supplementary Figures S5, S6.

**FIGURE 4:**
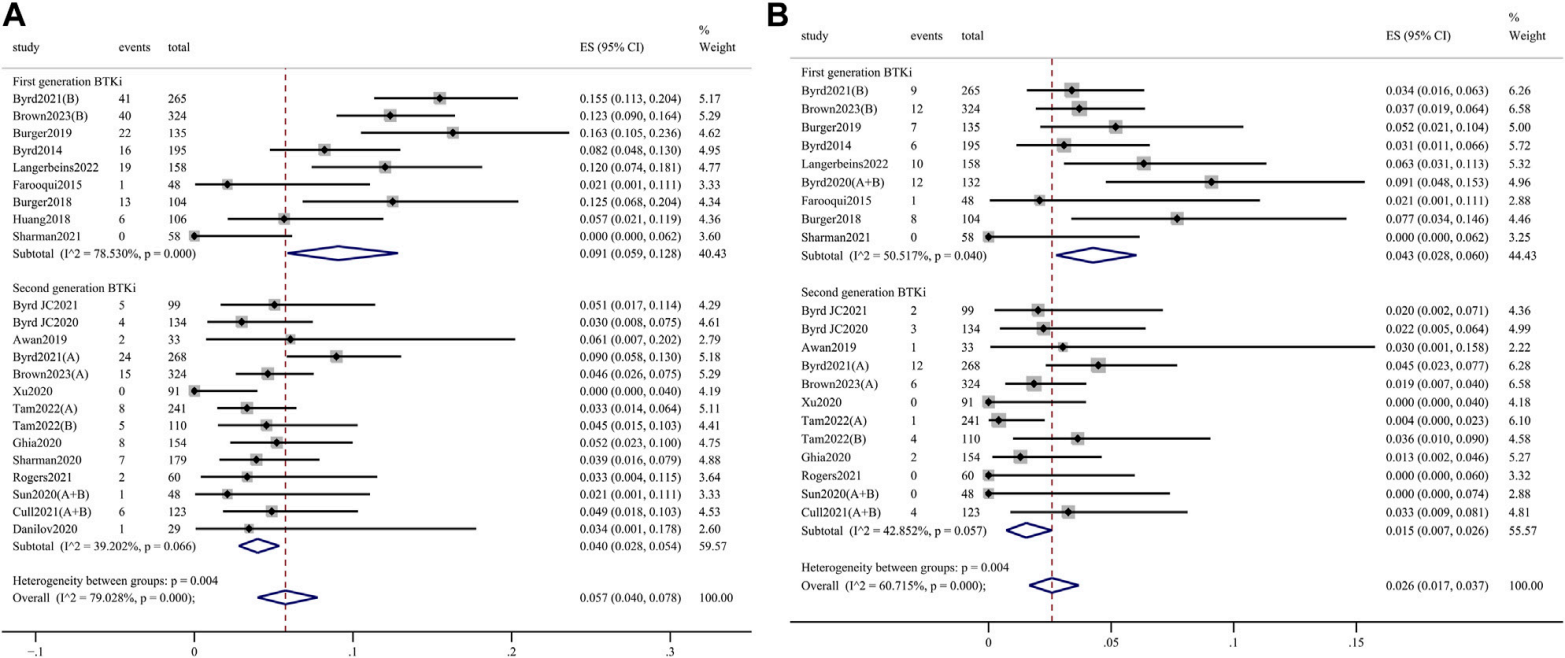
Forest plots for pooled incidences of any-grade atrial fibrillation **(A)** and grade 3 or higher atrial fibrillation **(B)**.

AEs of clinical interest, including atrial fibrillation, hypertension, bleeding, and infection events, were assessed to further explore the safety profile. Any-grade atrial fibrillation incidence was significantly higher in the 1st-generation BTKi group than in the 2nd-generation group (9.1%, 95% CI 5.9%–12.8% vs. 4.0%, 95% CI 2.8%–5.4%; *p* = 0.004) (Figure 4A). Meanwhile, grade 3 or higher atrial fibrillation incidence was significantly higher in the 1st-generation BTKi group than the 2nd-generation group (4.3%, 95% CI 2.8%–6.0% vs. 1.5%, 95% CI 0.7%–2.6%; *p* = 0.004) (Figure 4B). The pooled incidence of any-grade hypertension was 18.7% (95% CI 12.4%–26.0%) in the 1st-generation BTKi group and 12.1% (95% CI 8.1%–16.8%) in the 2nd-generation group. The pooled incidence of grade 3 or higher hypertension was 9.3% (95% CI 4.4%–15.9%) in the 1st-generation BTKi group and 5.3% (95% CI 3.1%–8.0%) in the 2nd-generation group. Compared to the 2nd-generation BTKi group, the incidences of any-grade and grade 3 or higher infections were higher in the 1st-generation group, with no statistically significant difference (any-grade: 73.6%, 95% CI 67.7%–79.1% vs. 67.7%, 95% CI 59.8%–75.1%; ≥3 grade: 28.3%, 95% CI 21.7%–35.4% vs. 20.9%, 95% CI 16.4%–25.7%). However, the pooled incidence of any-grade bleeding was lower, while grade 3 or higher was higher in the 1st-generation BTKi group than the 2nd-generation group. In addition to atrial fibrillation, the differences of hypertension, bleeding, and infection events were not significant. The meta-analysis results are shown in Supplementary Figures S7–S9.

## 4 Discussion

In this study, we found no statistical difference in the efficacy between 1st-generation and 2nd-generation BTKi. Therefore, we believed that the efficacy of the two generations of BTKi in the treatment of CLL was comparable. This was in accordance with the systematic review of clinical trials that reported data on the outcome of first- or second-generation BTKi for patients with Waldenström’s macroglobulinemia (Abushukair et al., 2022). In the first randomized phase Ⅲ trial comparing different selective BTKi, acalabrutinib demonstrated noninferior PFS compared to ibrutinib in patients with CLL (Byrd et al., 2021b). The overall response rate was 81% for acalabrutinib and 77% for ibrutinib. In the latest phase Ⅲ head-to-head trial, Brown et al. established the superiority of zanubrutinib by assessing PFS and ORR as key end points (Brown et al., 2023). The rates of 24-month PFS were 78.4% among patients who received zanubrutinib and 65.9% among patients who received ibrutinib. The ORR of patients in the zanubrutinib group was higher than in the inbrutinib group. Notably, the patients screened and assigned had relapsed or refractory CLL in these phase Ⅲ trials. We further divided the population into two subgroups, the R/R and TN groups, for meta-analysis.

In our subgroup analysis of relapsed or refractory patients, the ORR and 24-month PFS rates were significantly higher in the 2nd-generation BTKi group than in the 1st-generation group. To date, many clinical trials of 2nd-generation BTKi for R/R CLL patients have indeed achieved high response and survival rates. In the phase Ⅲ study of acalabrutinib monotherapy *versus* combination regimens, acalabrutinib exhibited improved PFS and similar ORR compared with the investigator’s choice (Ghia et al., 2020). In the acalabrutinib monotherapy group, the results demonstrated that ORR and 12-month PFS was 81% and 88%, respectively, for patients who previously received systemic therapy. The phase Ⅱ single-arm study of zanubrutinib showed 77 out of 91 Chinese patients with R/R CLL received a response (Xu et al., 2020). The single-agent phase Ⅱ trial (ACE-CL-208) affirmed that acalabrutinib was still effective in R/R CLL patients who were intolerant of ibrutinib (Rogers et al., 2021). Byrd et al. (2014) conducted the RESONATE trial to confirm ibrutinib as having a more significant effect than ofatumumab on previously treated CLL patients, but ultimately treatment achieved response in only 63% of ibrutinib-treated patients(Byrd et al., 2014). The 2nd-generation BTKi may be a suitable alternative to 1st-generation in CLL patients who may benefit from BTKi therapy, especially relapsed/refractory CLL patients.

No significant superiority of 2nd-generation BTKi was found in the treatment-naive CLL population. Despite this, TN patients had higher response and survival rates than R/R patients when they were treated with the same BTKi. The RESONATE and RESONATE-2 studies were two independent phase Ⅲ clinical trials of ibrutinib for patients with previously treated and untreated CLL, respectively (Byrd et al., 2014; Burger et al., 2015). At the same dosage (420 mg once daily) of inbrutinib, first-line treatment generated response in 92% of patients with TN CLL, while second- or post-line treatment generated response in 63% of patients with R/R CLL. Acalabrutinib was approved for patients with TN and R/R CLL based upon its efficacy in the ELEVATE-TN and ASCEND studies. Among 155 patients with R/R CLL in the phase Ⅲ ASCEND study, 126 (81%) achieved response after receiving acalabrutinib monotherapy at a dose of 100 mg twice daily (Ghia et al., 2020). The results of efficacy showed 12-month PFS was 88% and 12-month OS was 94% in the acalabrutinib monotherapy group. The ELEVATE-TN study demonstrated that acalabrutinib improved PFS over chemoimmunotherapy, supporting the use of acalabrutinib as a new treatment option for patients with TN CLL (Sharman et al., 2020). The results showed that 24-month PFS was 87% and overall response was 86% for acalabrutinib monotherapy. In the SEQUOIA study, Tam et al. (2022) evaluated the efficacy of zanubrutinib as front-line therapy in patients with CLL. They found that ORR was as high as 94.6%, and 24-month PFS was 85.5% in the zanubrutinib group (Tam et al., 2022). Another study estimated the PFS of patients with different prior lines of therapy, and the results reported that patients with fewer treatment lines had the advantage of longer PFS (Cull et al., 2022).

Both generations of BTKi have shown gratifying results in terms of overall survival and even long-term survival data. All studies included in our meta-analysis had rates of 12-month OS to be more than 90%, with the exception of the phase Ⅱ trial of orelabrutinib. Our meta-analysis calculated the pooled rates of 24-month OS in both generation BTKi remained at 90%. In a single-center trial of relapsed and treatment-naive high-risk CLL, OS at 36 months was 92% in patients receiving ibrutinib (Burger et al., 2019). With the median follow-up of 5 years, O'Brien et al. (2018) demonstrated the efficacy of ibrutinib for patients with CLL, with a 92% 5-year OS rate in TN and 60% in R/R patients (O'Brien et al., 2018). An up-to-8-year follow-up data from a RESONATE-2 study of ibrutinib reported that 59% of patients randomized to ibrutinib were alive (Barr et al., 2022). Long-term follow-up of ibrutinib in the RESONATE trial reported that median OS was 67.7 months in the ibrutinib group (Munir et al., 2019). Until now, 2nd-generation BTKi trials were conducted for a short period so that most have not yet reported long-term data. A phase Ⅱ study of acalabrutinib reported that 24-month and 36-month OS rates were 81% and 78%, respectively, for patients with CLL who were ibrutinib-intolerant (Rogers et al., 2021). Based on long-term follow-up from the phase Ⅰ/Ⅱ AU-003 study, zanubrutinib resulted in overall survival rates of 96% at 24 months and 91% at 36 months (Cull et al., 2022).

Ibrutinib has been approved for various B cell malignancies, but its adverse effects are still not negligible (Paydas, 2019). The common adverse effects of ibrutinib include diarrhea, neutropenia, bleeding, infection, and arthralgia, some of which may be caused by off-target kinase inhibition (Munir et al., 2019). In order to reduce the incidence of AEs, 2nd-generation BTKi was designed based on higher BTK specificity to achieve maximum BTK occupancy and minimize off-target binding (Guo et al., 2019). Our pooled results showed that the incidence of grade 3 or higher diarrhea was significantly higher with 1st-generation BTKi and that the incidence of any-grade headache was significantly higher with 2nd-generation BTKi, which was consistent with safety analyses in the ELEVATE-RR trial (Seymour et al., 2023). In terms of ibrutinib, the incidence of grade 3 or higher AEs was similar to that previously reported, at approximately 70% (Byrd et al., 2021b; Brown et al., 2023). The pooled incidences of grade 3 or higher AEs with 2nd-generation BTKi were lower than that reported at only 52% (Byrd et al., 2021b; Brown et al., 2023). As reported in a retrospective study, toxicity was the common reason for ibrutinib discontinuation (Mato et al., 2018). Acalabrutinib was still an option for patients who had previously discontinued ibrutinib due to adverse events, as the rate of acalabrutinib discontinuation due to adverse events in such patients was only 17% (Rogers et al., 2021). A phase Ⅱ study further demonstrated that patients who are intolerant to ibrutinib and acalabrutinib may continue to benefit from zanubrutinib treatment with few intolerance events (Shadman et al., 2023).

The adverse events of clinical interest included atrial fibrillation, hypertension, hemorrhage, and infections. Our meta-analysis showed that patients who received 2nd-generation BTKi had a significantly lower incidence of atrial fibrillation than patients who received 1st-generation BTKi, which is similar to the ELEVATE-RR and ALPINE studies (Byrd et al., 2021b; Brown et al., 2023). This may be associated with the hypothesis that 2nd-generation BTKi reduces the off-target kinase inhibition of C-terminal Src kinase to avoid the increased risk of cardiac adverse events (Guo et al., 2019). A pooled analysis of 762 patients with CLL who received acalabrutinib found that the rate of any-grade atrial fibrillation/flutter was 5% and hypertension was 9% (Brown et al., 2022). The comparison of hypertension AEs between the two generations of BTKi had different results in previous trials, including the high incidence of zanubrutinib in the ASPEN trial, the high incidence of ibrutinib in the ELEVATE trial, and similar incidence in the ALPINE trial (Tam et al., 2020; Byrd et al., 2021b; Brown et al., 2023). After statistical analysis, our study confirmed that hypertension AEs occurred more frequently in ibrutinib. A meta-analysis of bleeding risk associated with BTKi suggested that ibrutinib and acalabrutinib have a higher risk of bleeding compared to control drugs, and ibrutinib tended to increase the risk more than acalabrutinib (Jiang et al., 2022). Any-grade infections and ≥3 grade infections were comparable between the two generations of BTKi, with the most common infections being pneumonia, sepsis, and urinary tract infections (Byrd et al., 2021b).

Although we have completed this comprehensive review of efficacy and safety of BTKi, some limitations remain. First, the studies of tirabrutinib and orelabrutinib accounted for a relatively small proportion of the included studies and did not provide sufficient data for subgroup analysis of drug type. Second, due to a lack of reports on risk factors such as chromosome 17p deletion and TP53 mutation status, and chromosome 11q deletion status and IGHV mutational status, it was difficult to pool these factors in our analysis. Third, the follow-up time of the 2nd-generation BTKi trial was short, so the comparison of long-term efficacy in our study was insufficient.

## 5 Conclusion

In this first meta-analysis of BTKi therapy for CLL, 2nd-generation BTKi had comparable efficacy compared to 1st-generation, being only better in R/R patients with CLL. Compared to 1st-generation BTKi, 2nd-generation provided a significant difference of AEs, particularly in less than grade 3 or higher AEs, less than any-grade and grade 3 or higher atrial fibrillation, less than grade 3 or high diarrhea, and more than any-grade headache. Clinicians should consider these results and patients’ comorbidities when selecting 2nd-generation BTKi treatment regimen.

## Data Availability

The original contributions presented in the study are included in the article/Supplementary Material; further inquiries can be directed to the corresponding author.
