# Supplementary material for: Efficacy and safety of first- versus second-generation Bruton tyrosine kinase inhibitors in chronic lymphocytic leukemia: a systematic review and meta-analysis
Source: Front Pharmacol. 2024 Jul 10;15:1413985. doi: 10.3389/fphar.2024.1413985 (PMC11266288; doi:10.3389/fphar.2024.1413985)
Supplement: Supplementary file 1 [file DataSheet1.PDF]

Supplementary Table S1-4. and Figure S1-9.

Table S1. Search algorithm

Databases	Search	Results
**PubMed**	#1 ((((((((((((BTK[Title/Abstract]) OR (Bruton tyrosine kinase[Title/Abstract])) OR (Ibrutinib[Title/Abstract])) OR (Acalabrutinib[Title/Abstract])) OR (Zanubrutinib[Title/Abstract])) OR (Orelabrutinib[Title/Abstract])) OR (PCI-32765[Title/Abstract])) OR (CRA-032765[Title/Abstract])) OR (ACP-196[Title/Abstract])) OR (BGB-3111[Title/Abstract])) OR (ONO-4059[Title/Abstract])) OR (GS-4059[Title/Abstract])) OR (ICP-022[Title/Abstract])) OR (Tirabrutinib[Title/Abstract])	6,724
	#2 (CLL[Title/Abstract]) OR (Chronic Lymphocytic Leukemia[Title/Abstract])	24,804
	#3 "Clinical Trial"[Publication Type]	980,218
	#1 AND #2 AND #3	197
**Web of Science**	#1(((((((((((((TS=(BTK)) OR TS=(Bruton tyrosine kinase)) OR TS=(Ibrutinib)) OR TS=(Acalabrutinib)) OR TS=(Zanubrutinib)) OR TS=(Tirabrutinib)) OR TS=(orelabrutinib))) OR TS=(PCI-32765)) OR TS=(CRA-032765)) OR TS=(ACP-196)) OR TS=(BGB-3111)) OR TS=(ONO-4059)) OR TS=(GS-4059)) OR TS=(ICP-022)	11,135
	#2(TS=(CLL)) OR TS=(Chronic Lymphocytic Leukemia)	45.645
	#3(((((((TS=(clinical trial)) AND TS=(Clinical Trial, Phase I)) OR TS=(Clinical Trial,a Phase II)) OR TS=(Clinical Trial, Phase III)) OR TS=(Clinical Trial, Phase IV)) OR TS=(Randomized Controlled Trial)) OR TS=(Adaptive Clinical Trial)) OR TS=(Controlled Clinical Trial)	708,639
	#1 AND #2 AND #3	271
**Embase**	#1 btk:ti,ab,kw OR 'bruton tyrosine kinase':ti,ab,kw OR ibrutinib:ti,ab,kw OR acalabrutinib:ti,ab,kw OR zanubrutinib:ti,ab,kw OR tirabrutinib:ti,ab,kw OR orelabrutinib:ti,ab,kw OR 'pci 32765':ti,ab,kw OR 'cra 032765':ti,ab,kw OR 'acp 196':ti,ab,kw OR 'bgb 3111':ti,ab,kw OR 'ono 4059':ti,ab,kw OR 'gs 4059':ti,ab,kw OR 'icp 022':ti,ab,kw	13,859
	#2 cll:ti,ab,kw OR 'chronic lymphocytic leukemia':ti,ab,kw	43,126
	#3 'clinical trial':ti,ab,kw OR 'clinical trial, phase i':ti,ab,kw OR 'clinical trial, phase ii':ti,ab,kw OR 'clinical trial, phase iii':ti,ab,kw OR 'clinical trial, phase iv':ti,ab,kw OR 'randomized controlled trial':ti,ab,kw OR 'adaptive clinical trial':ti,ab,kw OR 'controlled clinical trial':ti,ab,kw	440,303
	#1 AND #2 AND #3	349

Table S2. Demographic and clinical characteristics in included trials

Study ID	Region	Median age (range), years	Male, no.(%)	Intervention	Dosage	NO. of prior therapies	ECOG scores
Rogers2021	23 centers in 6 countries	69.5(43-88)	38 (63%)	Acalabrutinib	100mg bid	2(1-10)	≤1 (97%)
Sun2020	United States	64(45-83)	/	Acalabrutinib	100mg bid, 200mg qd	0, 1(1-2)	/
Cull2021(A)	23 centers in 6 countries	66.0(24–87)	74 (73.3%)	Acalabrutinib	160mg bid, 320mg qd	2(1-10)	≤2
Cull2021(B)	23 centers in 6 countries	69.5(48–87)	18 (81. 8%)	Acalabrutinib	160mg bid, 320mg qd	0	≤2
Byrd2021(A)	124 centers in 15 countries	66(41-89)	185 (69.0%)	Acalabrutinib	100mg bid	2(1-9)	≤2
Ghia2020	25 countries	68(32-89)	108 (70%)	Acalabrutinib	100mg bid	/	≤2
Sharman2020	18 countries	70(66-75)	111 (62%)	Acalabrutinib	100mg bid	0	≤2
Byrd JC2020	United States, Italy, United Kingdom	66(42-85)	99 (74%)	Acalabrutinib	100 mg bid	2(1-13)	≤2
Byrd JC2021	United States, Italy, United Kingdom	64(33-85)	66 (67%)	Acalabrutinib	200 mg qd or 100 mg bid	0	≤2
Awan2019	United States, Italy, United Kingdom	64(50-82)	20 (61%)	Acalabrutinib	200 mg qd or 100 mg bid	4(2-13)	≤2
Byrd2021(B)	124 centers in 15 countries	65(28-88)	194 (73.2%)	Ibrutinib	420mg qd	2(1-12)	≤2
Brown2023(B)	15 countries	68(35-89)	232 (71.4%)	Ibrutinib	420mg qd	1(1-12)	/
Burger2019	16 countries	73(65–89)	88 (65%)	Ibrutinib	420mg qd	0	≤2

Byrd2014	67sites	67(30-86）	129（66%）	Ibrutinib	420mg qd	3(1-12)	≤2
Langerbeins2022	89 centers in Germany	64(38-85)	137 (75.3%)	Ibrutinib	420mg qd	0	≤2
Huang2018	29 sites in China, Australia, Taiwan, and Malaysia	65(39-87)	29 (27.4%)	Ibrutinib	420mg qd	1	≤1
Sharman2021	119 sites in the USA and Israel	67 (62–74)	46 (74%)	Ibrutinib	420mg qd	1 (1–2)	≤2
Farooqui2015	single center in US	62(33–82)	31 (60.8%)	Ibrutinib	420mg qd	/	/
Burger2018	single center in US	65(44-83)	75 (72.1%)	Ibrutinib	420mg qd	1(0-7)	≤1
Byrd2020(A)	10 sites in US，16 sites in US	64(37-82)	79 (78%)	Ibrutinib	420mg qd, 840mg qd	4 (1–12)	≤2
Byrd2020(B)	10 sites in US，16 sites in US	71(65-84)	19 (61%)	Ibrutinib	420mg qd, 840mg qd	0	≤2
Byrd2013	8 centers	66(37-82)	65 (76%)	Ibrutinib	420mg qd, 840mg qd	4(1-12)	≤2
Danilov2020	United States, the United Kingdom, and France	70 (52–91)	17 (58.6%)	Tirabrutinib	80 mg qd	1 (1–6)	≤2
Walter2016	France, United Kingdom	67(40-83)	23 (82.1%)	Tirabrutinib	20 to 600 mg qd	3.5(2-7)	/
Brown2023(A)	15 countries	67(35-90）	213（65.1）	zanubrutinib	160mg bid	1(1-6)	/
Tam2022(A)	14 countries	70(66-75)	154 (64%)	zanubrutinib	160mg bid	0	≤2
Tam2022(B)	14 countries	70(66-74)	79 (71%)	zanubrutinib	160mg bid	0	≤2
Xu2020	China	61(35-87)	52 (57.1%)	zanubrutinib	160mg bid	1(1-9)	≤2
Xu2023	China	60(36–78)	51 (63.8%)	Orelabrutinib	150 mg qd	1(1-7)	≤2

Table S3. The MINORS item score for non-comparative studies

study ID	Q1	Q2	Q3	Q4	Q5	Q6	Q7	Q8	Score
Rogers2021	2	2	2	2	0	2	2	0	12
Sun2020	2	2	2	2	0	2	2	2	14
Farooqui2015	2	2	2	2	0	2	2	2	12
Xu2020	2	2	2	2	2	2	2	2	16
Xu2023	2	2	2	2	2	2	2	2	16
Byrd2020	2	2	2	2	0	2	2	0	12
Danilov2020	2	2	2	2	0	2	2	0	12
Byrd JC2021	2	2	2	2	0	2	2	0	12
Cull2021	2	2	2	2	0	2	2	2	14
Awan2019	2	2	2	2	0	2	2	0	12
Walter2016	2	2	2	2	0	2	2	0	12
Byrd2013	2	2	2	2	0	2	2	2	14
Byrd JC2020	2	2	2	2	0	2	2	0	12

The items are scored 0 (not reported), 1 (reported but inadequate) or 2 (reported and adequate).

Table S4. Long-term survival data reported in included studies

BTKi Generation	Study	Intervention	Overall Survival	Progression-Free Survival
2nd generation	Rogers2021	Acalabrutinib	36-month: 78.3%	36-month: 58.3%
	Cull2021(B)	Acalabrutinib	36-months: 91%	36-month: 83%
	Byrd JC2021	Acalabrutinib		48-month: 96%
	Byrd JC2020	Acalabrutinib		48-month: 62%
1st generation	Burger2019	Ibrutinib	60-month: 83%<br>84-month: 78%	60-month: 70%<br>84-month: 59%
	Byrd2014	Ibrutinib	36-months:74%	36-month: 59%<br>60-month: 40%
	Byrd2020(A)	Ibrutinib	60-month: 60%<br>84-month: 55%	60-month: 44%<br>84-month: 34%
	Byrd2020(B)	Ibrutinib	60-month: 92%<br>84-month: 84%	60-month: 92%<br>84-month: 83%
	Burger2018	Ibrutinib	36-month: 92%	36-month: 86%

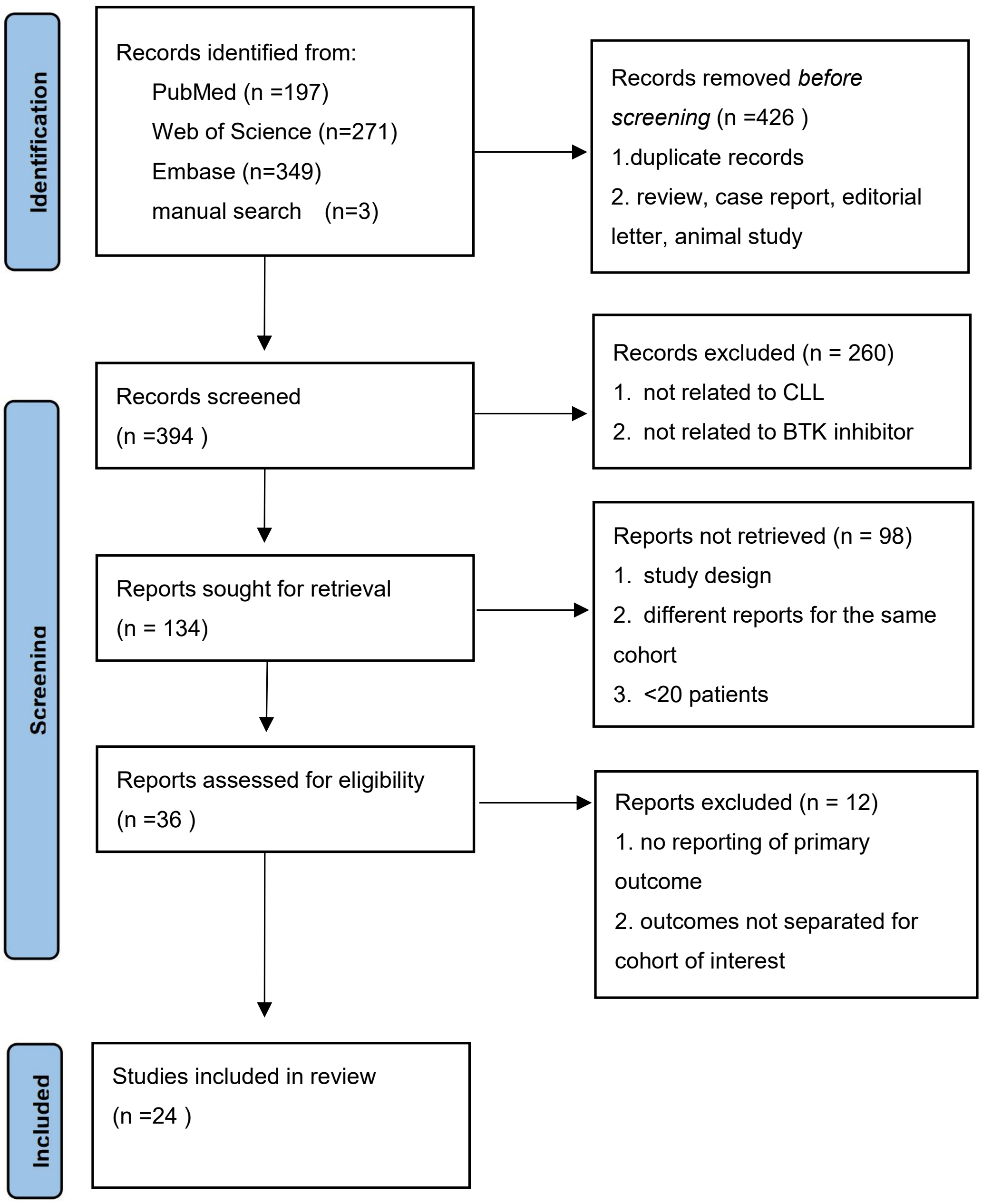

Figure S1. Flowchart diagram of literature search and screening.

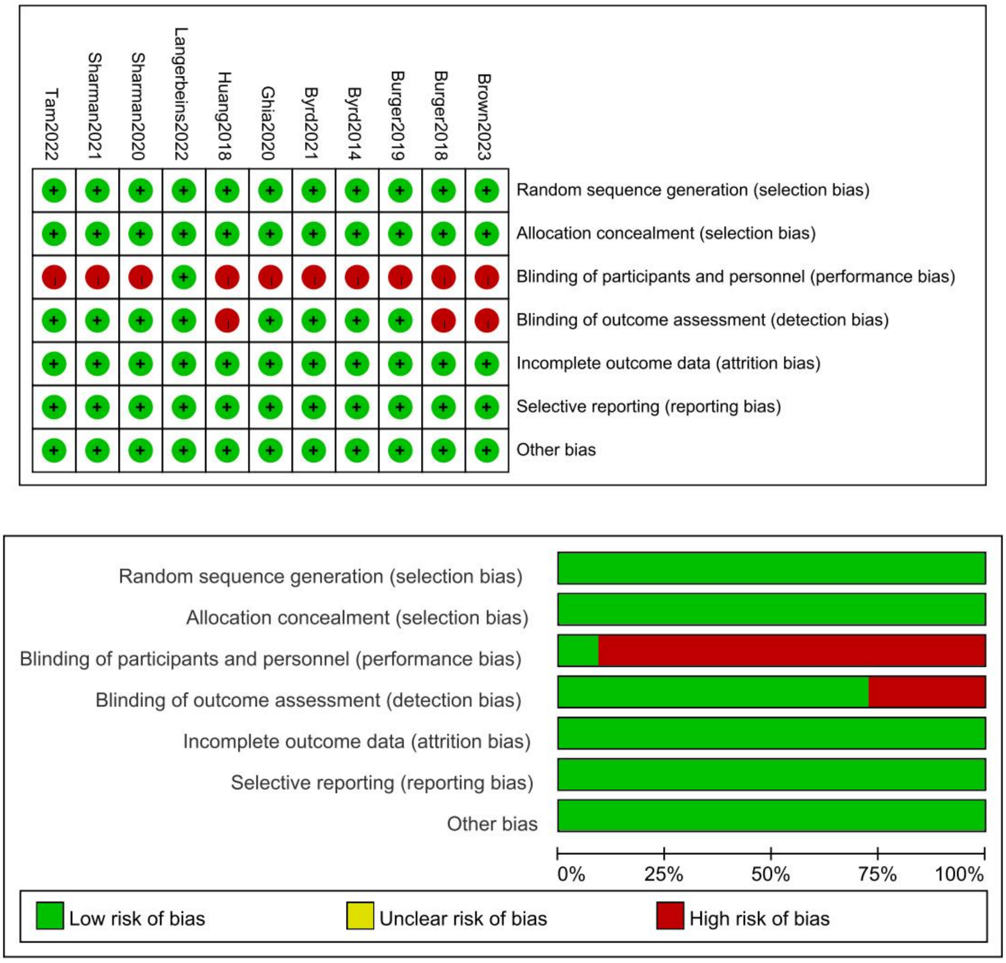

Figure S2. Traffic light plots and bar plots for risk of bias assessment in randomized controlled trails.

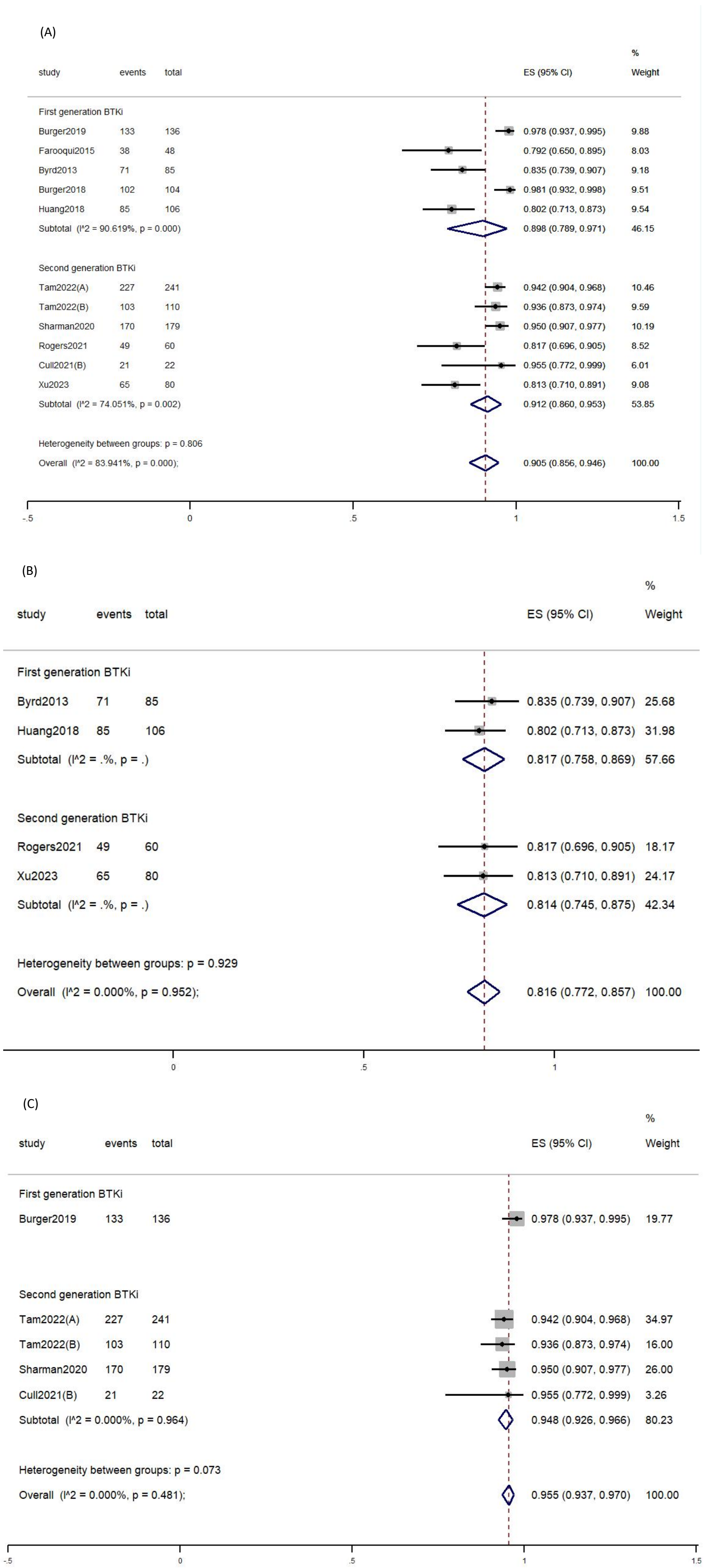

Figure S3. Forest plots for pooled 24-month OS in all patients (A), R/R patients (B) and TN patients (C).

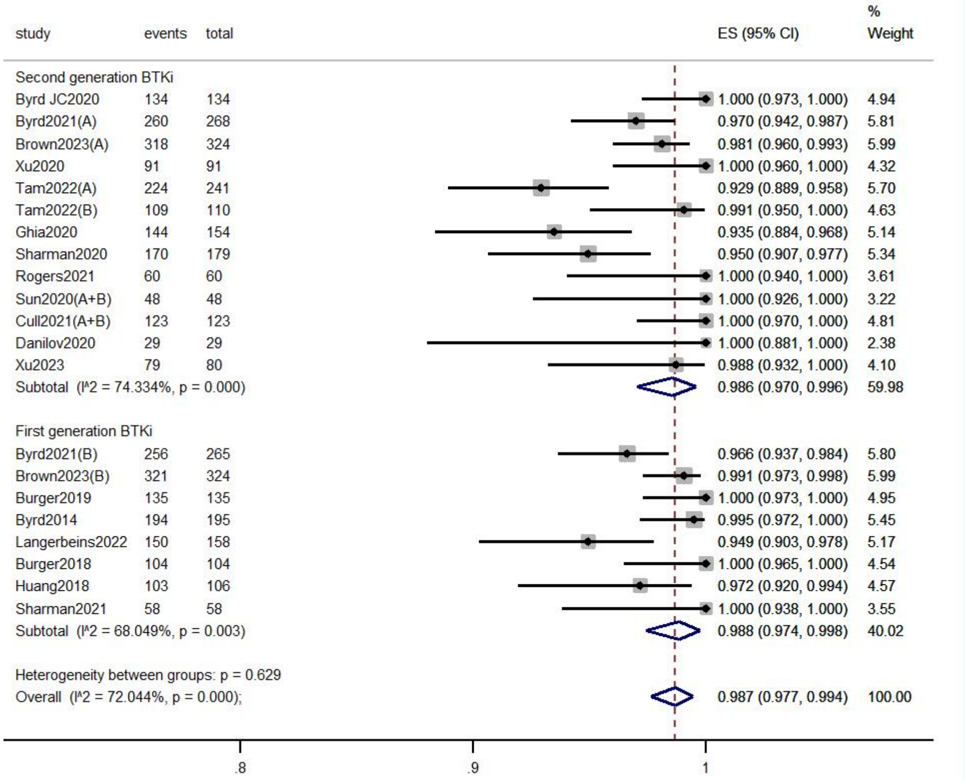

Figure S4. Forest plots for pooled incidences of any-grade adverse events.

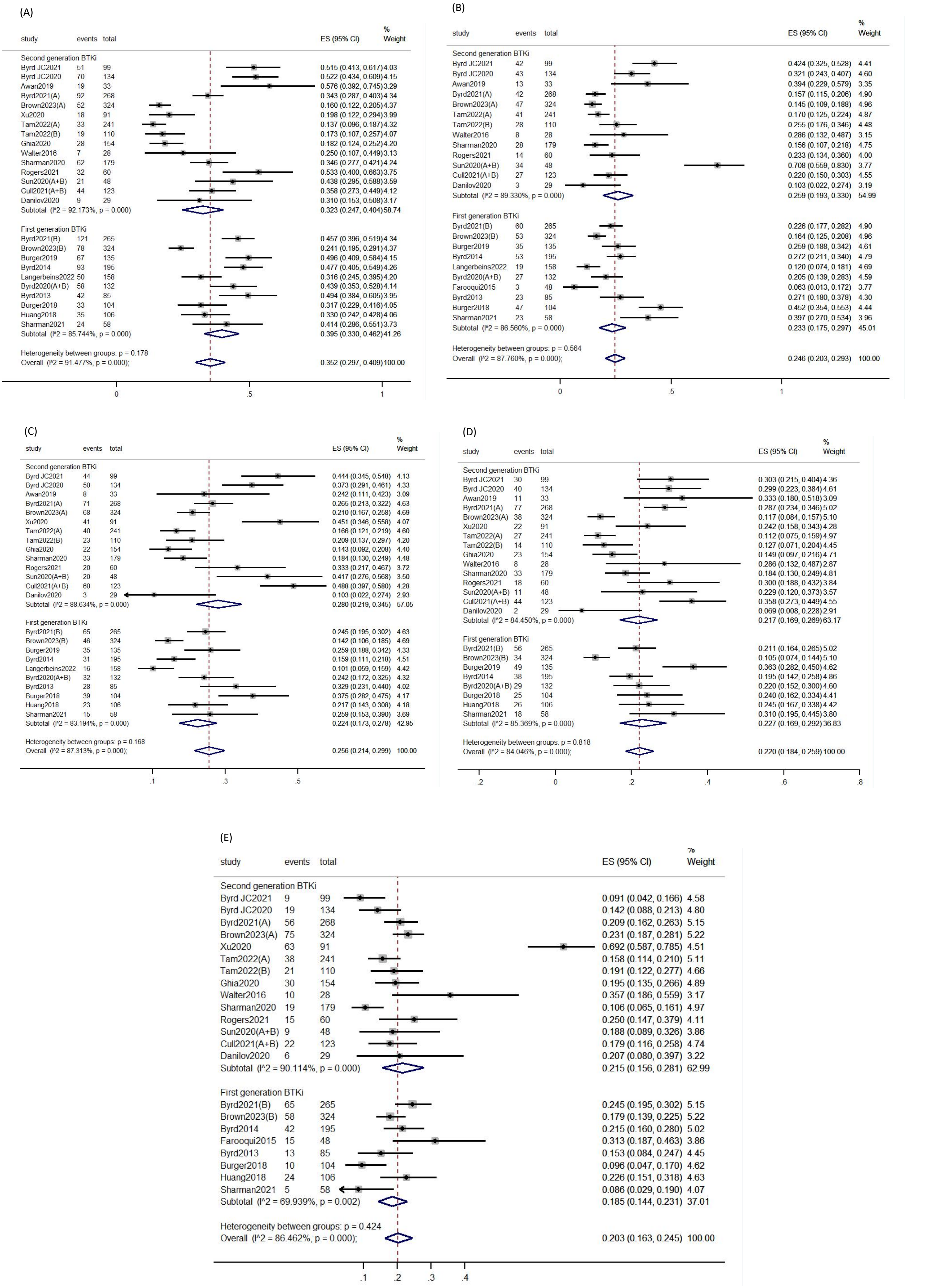

Figure S5. Forest plots for pooled incidences of any-grade diarrhoea (A), arthralgia/myalgia (B), upper respiratory tract infections (C), cough (D), neutropenia (E).

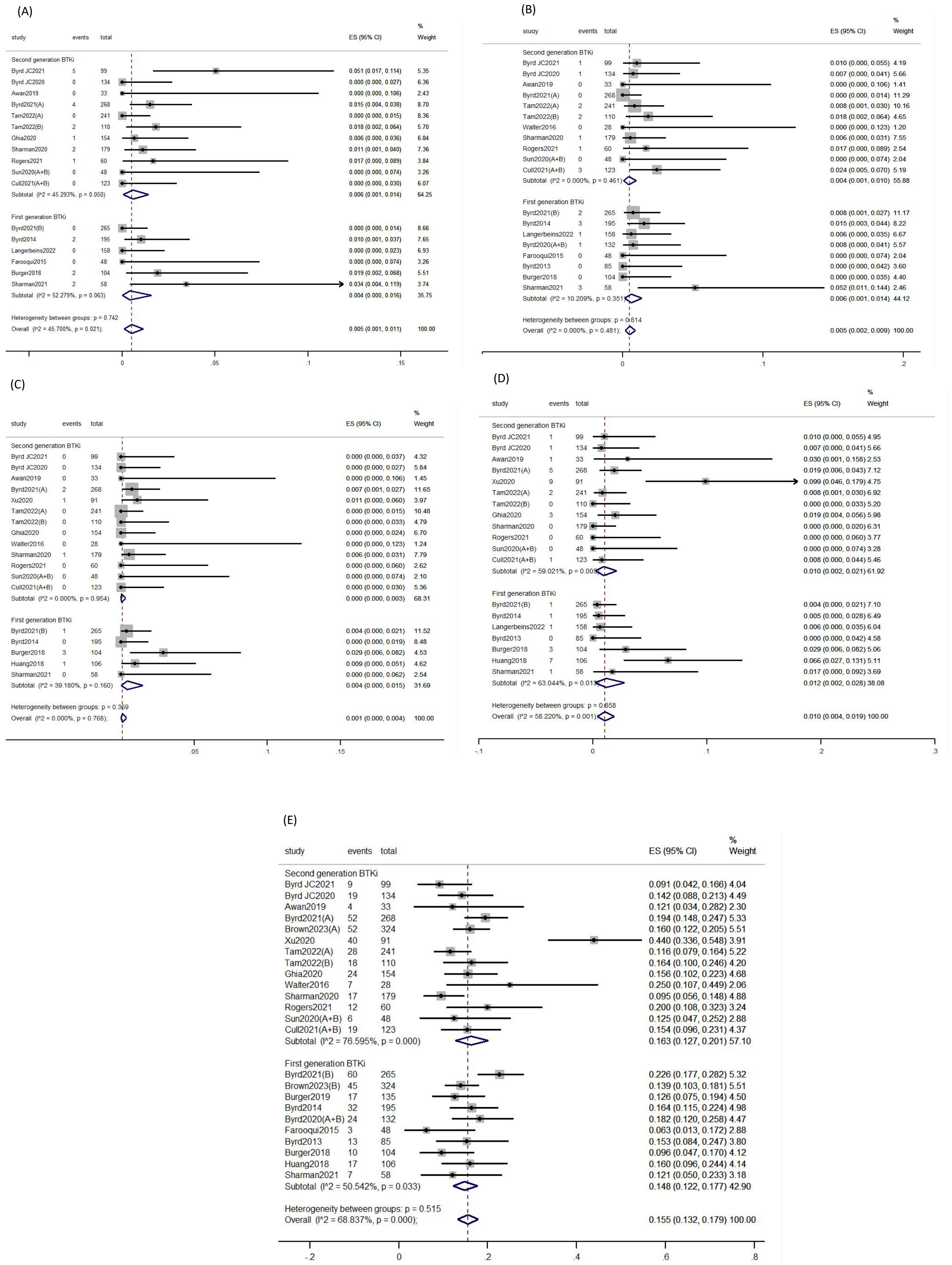

Figure S6. Forest plots for pooled incidences of grade 3 or high headache (A), arthralgia/myalgia (B), cough (C), upper respiratory tract infections (D), neutropenia (E).

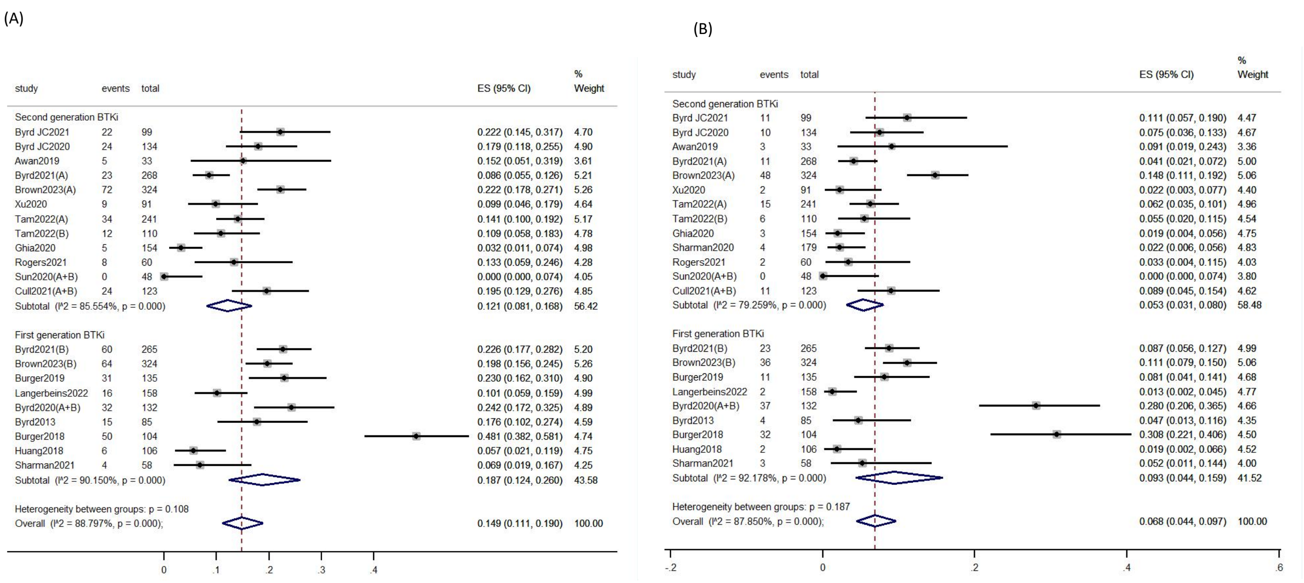

Figure S7. Forest plots for pooled incidences of any-grade hypertension (A), grade 3 or high hypertension (B).

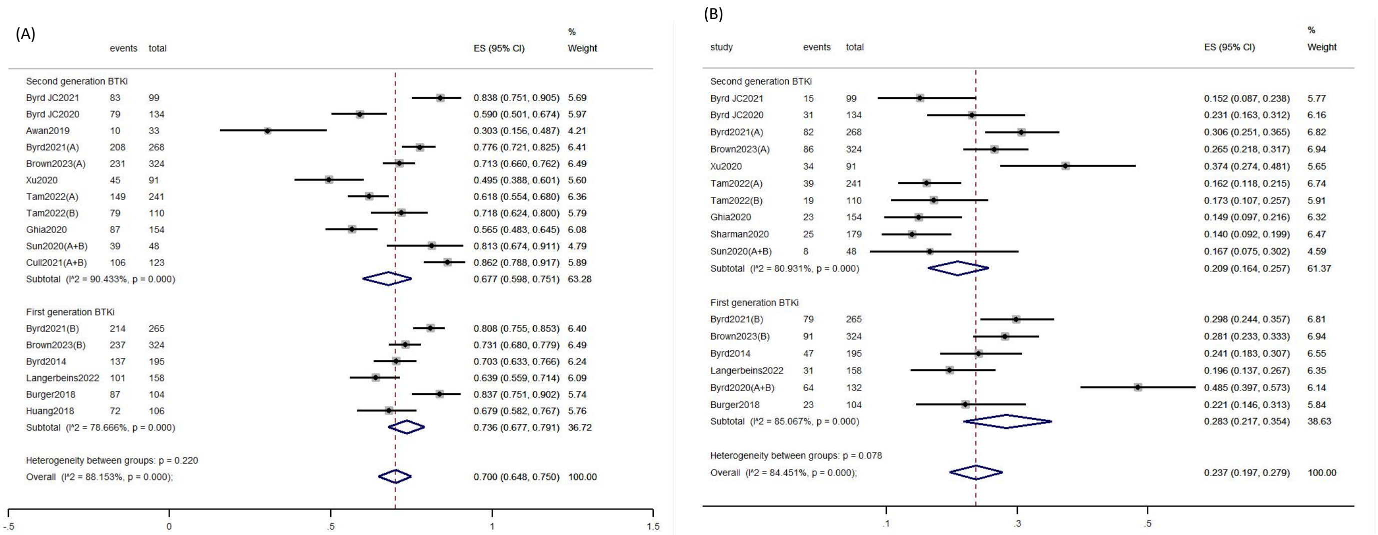

Figure S8. Forest plots for pooled incidences of any-grade infection (A), grade 3 or high infections (B) .

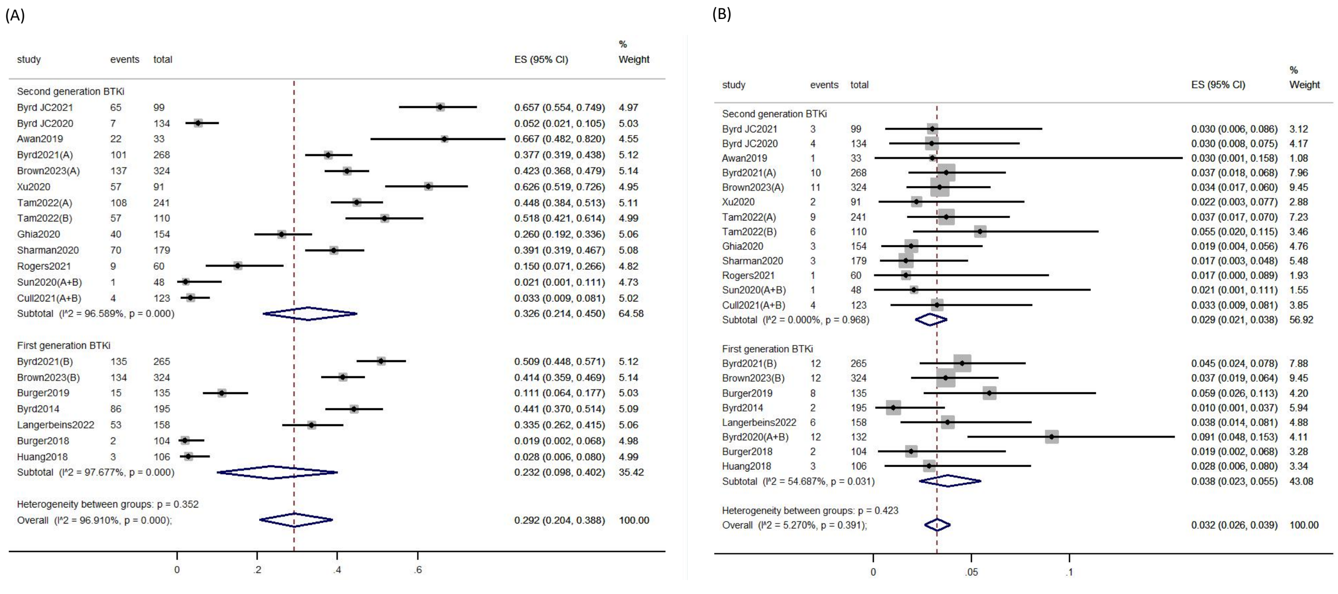

Figure S9. Forest plots for pooled incidences of any-grade bleeding (A), grade 3 or high bleeding (B).
